# Three new *Hutufeideria* Hirschmann & Hiramatsu, 1977 from Southeast Asia, with the description of Hutufeideriidae fam. nov. (Acari, Mesostigmata)

**DOI:** 10.3897/zookeys.1280.188887

**Published:** 2026-05-20

**Authors:** Jenő Kontschán, Rita Ábrahám, Sergey G. Ermilov

**Affiliations:** 1 Plant Protection Institute, HUN-REN Centre for Agricultural Research, Martonvásár, Hungary Plant Protection Institute, HUN-REN Centre for Agricultural Research Martonvásár Hungary; 2 Department of Plant Sciences, Albert Kázmér Faculty of Mosonmagyaróvár, Széchenyi István University, Mosonmagyaróvár, Hungary Institute of Environmental and Agricultural Biology (X-BIO), University of Tyumen Tyumen Russia; 3 Institute of Environmental and Agricultural Biology (X-BIO), University of Tyumen, Tyumen, Russia Albert Kázmér Faculty of Mosonmagyaróvár, Széchenyi István University Mosonmagyaróvár Hungary

**Keywords:** Indonesia, Malaysia, mites, morphology, new family, new species, systematics, taxonomy, Uropodina

## Abstract

Three new species of the genus *Hutufeideria* are described from the Oriental region. The first species, *H. perakensis***sp. nov**., was collected in Malaysia and differs from its congeners in the presence of a strongly sclerotized furrow on the dorsal shield and very long setae on the central area of the dorsal shield. The second species, *H. sarawakensis***sp. nov**., was found in Sarawak (Malaysia) and differs from other species of the genus in the shape of the setae on the caudal part of the dorsal shield and the shape of the female genital shield. The third species, *H. sumatraensis***sp. nov**., was collected from Sumatra (Indonesia) and has a tile-like sculptural pattern on the ventral shield; the dorsal shield is covered with large and irregular pits, and bears serrate setae on the caudal margins of the dorsal shield; these represent a unique character combination within the genus. Hutufeideriidae**fam. nov**. is diagnosed, with *Hutufeideria* as the type genus. A list of known species is provided.

## Introduction

The genus *Hutufeideria* was established by Hirschmann & Hiramatsu, (1977) for the species *H. hutuae* Hirschmann & Hiramatsu, 1977 and *H. feideri* Hirschmann & Hiramatsu, 1977 collected in New Guinea. Over the next few years, some new species were described from this genus from New Guinea (Hiramatsu 1978a, 1980, 1981), Japan (Hiramatsu 1979) and Indonesia (Hiramatsu 1983). More than 25 years later, Kontschán (2011) transferred *Kaszabjbaloghia hirschmanni* Hiramatsu, 1978 (see Hiramatsu 1978b) into the genus *Hutufeideria*, but he did not mention that the Australian *Hutufeideria hirschmanni* (Hiramatsu, 1978) (see Hiramatsu 1978b) is a homonym of the New Guinean *Hutufeideria hirschmanni* Hiramatsu, 1978 (see Hiramatsu 1978a). The two species were described simultaneously; therefore, Kontschán (2011), as the first reviser, proposed the following replacement name for the Australian species: *Hutufeideria hirschmannoides* Kontschán, 2011. Besides this nomenclature act, Kontschán (2011) described three new *Hutufeideria* species from Thailand. Five years later, Kontschán and Ripka (2016) also described another new species from Singapore.

Hirschmann (1979) revised his previous system (Gangsystematik der Parasitiformes) and introduced several new families within Uropodina, including Hutufeideriidae Hirschmann, 1979. These families were simply listed, with no formal description, diagnosis, or designation of a type genus. Halliday (2016) noted that Hirschmann’s family names are nomina nuda, but he suggested maintaining the original names and formally establishing them as a new family. These had been done with many other Hirschmann’s families (see Kontschán 2017; Kontschán and Friedrich 2017; Kontschán and Ermilov 2023 and Kontschán et al. 2023), and following this process, we establish a new family for the members of Hirschmann’s hutufeideriid mites in the present paper together with the description of three new *Hutufeideria* species found during the visits of the first author to Geneva’s Natural History Museum between 2013 and 2019.

## Material and methods

The examined *Hutufeideria* specimens were cleared in lactic acid and investigated on half-covered excavated slides. Illustrations were made with the aid of a drawing tube on a Leica DM 1000 scientific microscope. All specimens are stored in 75% ethanol and deposited in the Natural History Museum in Geneva. Abbreviations: ***h*** = hypostomal setae, ***st*** = sternal setae, ***lf*** = lyriform fissure, ***s*** = setae-like sensory organ, ***v*** = ventral setae. All measurements and the scales in the figures are given in micrometres (μm). The gnathosomal region is very similar to that seen in all *Hutufeideria* species; therefore, because the number of available specimens of the third species was very limited, we refrained here from breaking the idiosoma to study the gnathosomal part.

## Systematics

### Hutufeideriidae fam. nov.

Taxon classificationAnimaliaEchinostelialesHutufeideriidae

37F99E36-9F43-59DA-8F15-2F3EBA10960D

https://zoobank.org/502F8E18-5480-42C5-91A1-CE13FEA6CF7C

Hutufeideriidae Hirschmann, 1979: 69 (nomen nudum).
Hutufeideriidae —Halliday 2016: 353.

#### Type genus.

*Hutufeideria* Hirschmann & Hiramatsu, 1977: 69, by monotypy.

#### Diagnosis.

Idiosoma oval or rectangular, with brown coloration. Postdorsal shield present with weakly sclerotized, membranous caudal appendix. Genital shield of female scutiform or linguliform. Tritosternum with narrow basis, tritosternal laciniae subdivided into 3–4 pilose branches. Corniculi narrow, horn-like and bearing 1–5 lateral spines. Insertion of corniculi situated below level of insertion of *h1*. Internal malae pilose marginally. Hypostomal setae *h1* long, smooth or subdivided into 2–4 branches, *h2*, *h3* and *h4* marginally serrate. Epistome long, simple or apically subdivided into two branches, basally serrate and apically pilose. Chelicerae with internal sclerotised node, digitus fixus and digitus mobilis very long, with only 1–2 teeth apically. Palp trochanter with a short and a long marginally serrate or pilose ventral setae. Leg I with small apical claws.

#### Distribution.

Australia, Indonesia (Borneo and Sumatra), Malaysia (Perak and Sarawak), New Guinea, Japan, Singapore and Thailand.

#### Remarks.

When Hirschmann (1979) introduced the family name, Hutufeideriidae, he simply listed it and did not present a formal description, diagnosis, or designation of a type genus. Following Halliday’s (2016) suggestion about Hirschmann’s family name being a nomen nudum, we maintain the original name but formally establish it here as a new family. Hutufeideriidae is a monotypic family with one genus.

### Genus Hutufeideria

Taxon classificationAnimaliaEchinostelialesUropodidae

Hirschmann & Hiramatsu, 1977

32729210-B86B-528D-8C2E-0185BA615F58

Hutufeideria Hirschmann & Hiramatsu, 1977: 69.
Hutufeideria —Kontschán 2011: 58, Halliday 2015: 116, Kontschán 2024: 103–104.

#### Diagnosis.

See the family.

#### Type species.

*Hutufeideria hutuae* Hirschmann & Hiramatsu, 1977, by original designation.

#### Remarks.

Thus far, we know only 14 *Hutufeideria* species, all collected in the Austral-Asian and Southeast Asian regions.

##### List of the known *Hutufeideria* species

***Hutufeideria alata* Kontschán, 2011**

*Hutufeideria alata* Kontschán, 2011: 62–65.

*Hutufeideria alata*: Kontschán and Ripka 2016: 300; Kontschán 2024: 103.

**Distribution**. Thailand.

***Hutufeideria aoki* Hiramatsu, 1979**

*Hutufeideria aoki* Hiramatsu, 1979: 88–89

*Hutufeideria aoki*: Wiśniewski and Hirschmann 1993: 29; Kontschán 2011: 65; Kontschán and Ripka 2016: 300; Kontschán 2024: 103.

**Distribution**. Japan.

***Hutufeideria deliciosa* Hiramatsu, 1978**

*Hutufeideria deliciosa* Hiramatsu, 1978a: 108–109.

*Hutufeideria deliciosa*: Wiśniewski and Hirschmann 1993: 30; Kontschán 2011: 65; Kontschán and Ripka 2016: 300; Kontschán 2024: 103.

**Distribution**. New Guinea.

***Hutufeideria feideri* Hirschmann & Hiramatsu, 1977**

*Hutufeideria feideri* Hirschmann & Hiramatsu, 1977: 70–71.

*Hutufeideria feideri*: Wiśniewski and Hirschmann 1993: 30; Kontschán 2011: 65; Kontschán and Ripka 2016: 300; Kontschán 2024: 103.

**Distribution**. New Guinea.

***Hutufeideria feiderisimilis* Hiramatsu, 1981**

*Hutufeideria feiderisimilis* Hiramatsu, 1981: 104–105.

*Hutufeideria feiderisimilis* Hiramatsu, 1981: Wiśniewski and Hirschmann 1993: 30; Kontschán 2011: 65; Kontschán and Ripka 2016: 300; Kontschán 2024: 103.

**Distribution**. New Guinea.

***Hutufeideria haradai* Hiramatsu, 1983**

*Hutufeideria haradai* Hiramatsu, 1983: 66–68.

*Hutufeideria haradai*: Wiśniewski and Hirschmann 1993: 30; Kontschán 2011: 65; Kontschán and Ripka 2016: 300; Kontschán 2024: 103.

**Distribution**. Indonesia, Borneo.

***Hutufeideria hirschmanni* Hiramatsu, 1978**

*Hutufeideria hirschmanni* Hiramatsu, 1978a: 106–108.

*Hutufeideria hirschmanni*: Wiśniewski and Hirschmann 1993: 30; Kontschán 2011: 65; Kontschán and Ripka 2016: 300; Kontschán 2024: 103.

**Distribution**. New Guinea.

***Hutufeideria hirschmannoides* Kontschán, 2011**

*Kaszabjbaloghia hirschmanni*Hiramatsu 1978b: 109–111.

*Hutufeideria hirschmannoides* Kontschán, 2011: 58.

*Kaszabjbalogbia hirschmanni*: Wiśniewski and Hirschmann 1993: 32.

*Hutufeideria hirschmannoides*: Kontschán 2011: 65; Kontschán and Ripka 2016: 300; Kontschán 2024: 103.

**Distribution**. Australia.

***Hutufeideria hirschmannisimilis* Hiramatsu, 1980**

*Hutufeideria hirschmannisimilis* Hiramatsu, 1980: 50.

*Hutufeideria hirschmannisimilis*: Wiśniewski and Hirschmann 1993: 30; Kontschán 2011: 65; Kontschán and Ripka 2016: 300; Kontschán 2024: 103.

**Distribution**. New Guinea.

***Hutufeideria hutuae* Hirschmann & Hiramatsu, 1977**

*Hutufeideria hutuae* Hirschmann & Hiramatsu, 1977: 69–70.

*Hutufeideria hutuae*: Wiśniewski and Hirschmann 1993: 30; Kontschán 2011: 65; Kontschán and Ripka 2016: 300; Kontschán 2024: 103.

**Distribution**. New Guinea.

***Hutufeideria phuketensis* Kontschán, 2011**

*Hutufeideria phuketensis* Kontschán, 2011: 59–61.

*Hutufeideria phuketensis*: Kontschán and Ripka 2016: 300; Kontschán 2024: 103.

**Distribution**. Thailand.

***Hutufeideria singaporensis* Kontschán & Ripka, 2016**

*Hutufeideria singaporensis* Kontschán & Ripka, 2016: 292.

**Distribution**. Singapore.

**Note**. This species is missing from Kontschán (2024).

***Hutufeideria thailandica* Kontschán, 2011**

*Hutufeideria thailandica* Kontschán, 2011: 59.

*Hutufeideria thailandica*: Kontschán and Ripka 2016: 300; Kontschán 2024: 103.

**Distribution**. Thailand.

***Hutufeideria virtuosa* Hiramatsu, 1983**

*Hutufeideria virtuosa* Hiramatsu, 1983: 68–70.

*Hutufeideria virtuosa*: Wiśniewski and Hirschmann 1993: 30; Kontschán 2011: 65; Kontschán and Ripka 2016: 300; Kontschán 2024: 103.

**Distribution**. Indonesia, Borneo.

### Hutufeideria perakensis sp. nov.

Taxon classificationAnimaliaEchinostelialesUropodidae

D04FDC9B-4D12-569C-BDEA-FF82BC0B09C6

https://zoobank.org/99474DC4-801F-4B55-B16A-8A2433B072B4

Figs 1, 2, 3

#### Material examined.

***Holotype***. • Female, W-Malaysia, Perak, Maxwell Hill, 10 km E Taiping, 1200 m a.s.l., 4°52'N, 100°49'E, 22 December 2004, A. Schulz coll. ***Paratype***. • One male, same data as holotype.

#### Description.

**Female**. Length of idiosoma 1015, width 819 (*N* = 1). Shape oval, dorsally domed and strongly sclerotized.

**Figure 1.**
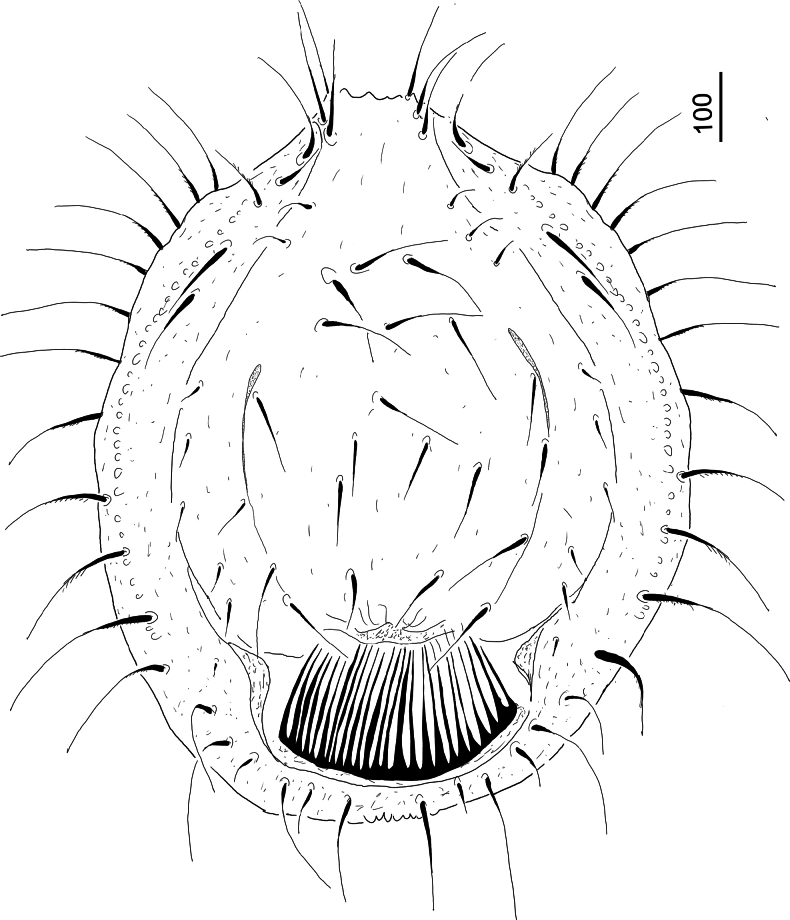
*Hutufeideria perakensis* sp. nov., dorsal view of female holotype.

***Dorsal idiosoma*** (Fig. 1). Dorsal and marginal shields completely separated. Central area of dorsal shield bordered by narrow strongly sclerotized furrows. Dorsal setae on central area long (c. 108–144) and smooth. Other dorsal setae close to margin of dorsal shield short (c. 45–54) and smooth. Dorsal shield without sculptural pattern. Marginal shield bearing c. 20 pairs of long (c. 155–194) and marginally pilose and three pairs of short (c. 45–64) and smooth setae. Marginal shield covered by oval pits on its surface. Postdorsal shield present, with a large, weakly sclerotized and deeply incised membranous caudal appendix.

**Figure 2.**
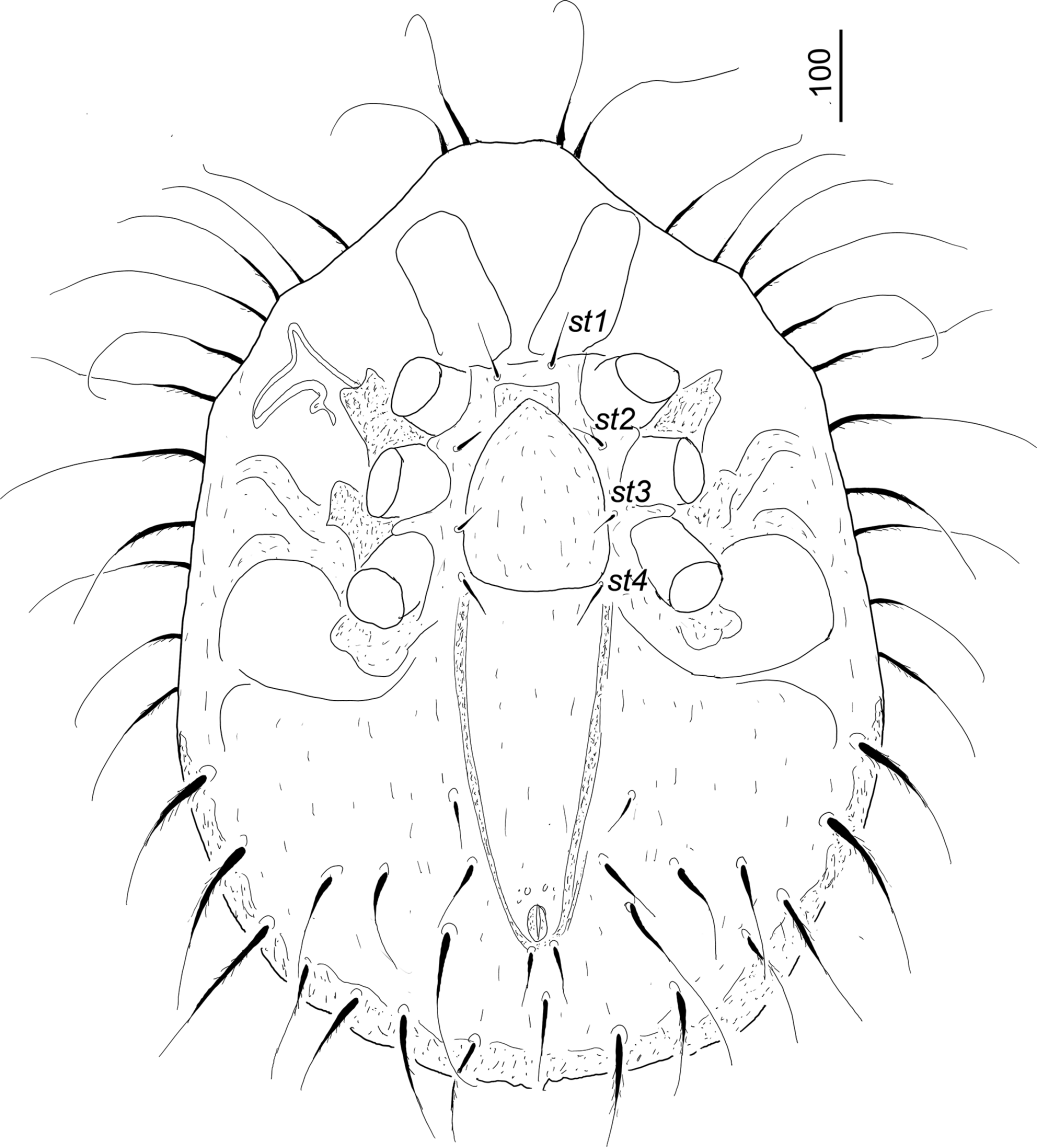
*Hutufeideria perakensis* sp. nov., ventral view of female holotype.

**Figure 3.**
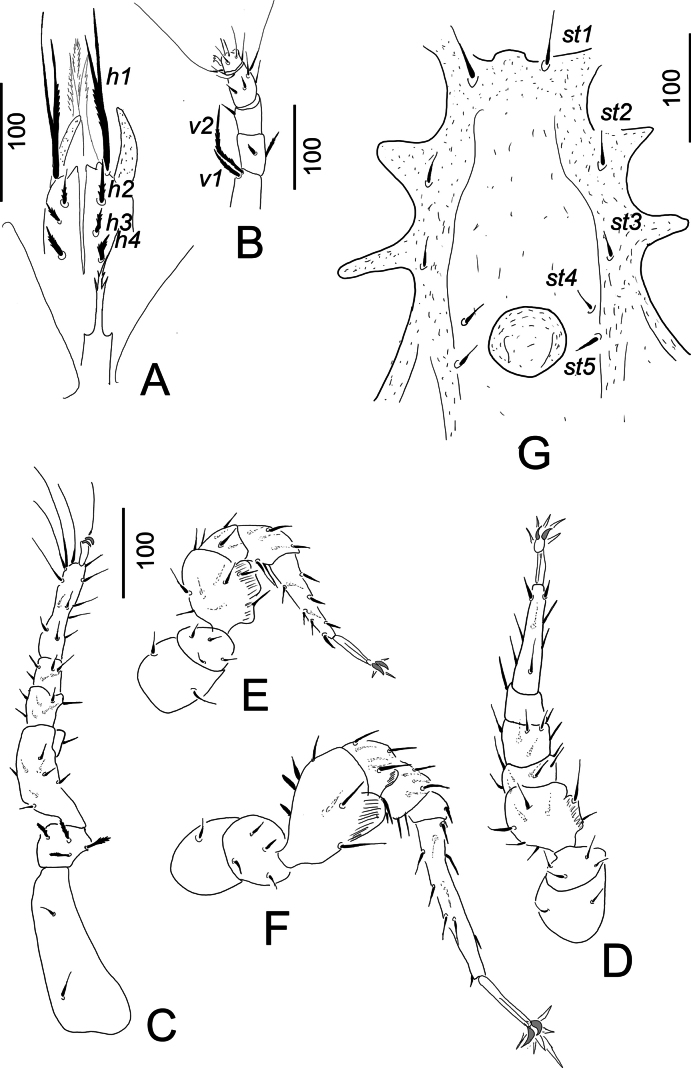
*Hutufeideria perakensis* sp. nov., female holotype (**A–F**), male paratype (**G**). **A**. Tritosternum and ventral view of gnathosoma and palp; **B**. Lateral view of palp; **C**. Ventrolateral view of leg I; **D**. Ventrolateral view of leg II; **E**. Ventrolateral view of leg III; **F**. Ventrolateral view of leg IV; **G**. Intercoxal area.

***Ventral idiosoma*** (Fig. 2). Surface of sternal shield smooth. Four smooth and needle-like sternal setae present. Setae *st1* (c. 60–61) situated close to anterior margin of sternal shield, *st2* (c. 38–40) at level of anterior margins of coxae III, *st3* (c. 30–31) at level of posterior margin of coxae III, *st4* (c. 50–51) at level of posterior margin of coxae IV. A V-shaped and strongly sclerotized furrow situated between *st4* and anal opening. Ventral shield without sculptural pattern. First pair of ventral setae short (c. 43–44), the other five pairs longer (c. 85–125). One pair of smooth adanal setae shorter (c. 62) than postanal seta (c. 90). Anal opening rounded, 40 long and 22 wide. One pair of pore-like structures situated anterior to anal opening. Pedofossae deep and their surface smooth, without separated furrows for tarsi IV. Genital shield scutiform (c. 200 long and 160 wide at level of *st3*), anteriorly slightly peaked, its surface without sculptural pattern. Stigmata situated between coxae II and III, peritremes M-shaped. Tritosternum with narrow basis, laciniae subdivided into three smooth branches (Fig. 3A).

***Gnathosoma*** (Fig. 3A). Corniculi long and horn-like, without lateral spines, internal malae longer than corniculi and marginally pilose. Hypostomal setae *h1* (c. 110–115 µm) subdivided into two branches of which one smooth and other one marginally pilose, *h2*, *h3* and *h4* marginally serrate and short (c. 23–35 µm). Epistome apically pilose. Palp trochanter with a short (*v1*) and a long (*v2*) marginally serrate seta, setae on femora serrate, other setae on palp smooth (Fig. 3B). Chelicerae not clearly visible.

***Legs*** (Fig. 3C–F). Majority of setae on legs smooth, but some setae pilose. Claw of leg I smaller than on others, flap-like prolongation visible on femora of leg I–IV. Length of legs (from the basis of coxae to the tip of tarsi): leg I 530–532, leg II 340–343, leg III 400–403, leg IV 510–512.

**Male**. Length of idiosoma 1010, width 810 (*N* = 1). Shape oval, posterior margin rounded.

***Dorsal side***. Ornamentation and chaetotaxy as in female.

***Ventral side***. Ornamentation and chaetotaxy of ventral shield as in female. Sternal shield without sculptural pattern, the positions of five pairs of smooth setae illustrated in Fig. 3G. Setae *st1* longer (c. 53–55), than others (c. 21–30) sternal setae. Genital shield rounded (63 long and 73 wide) and situated between coxae IV.

Nymphs and larvae unknown.

#### Etymology.

The name of the new species refers to the country where it was collected.

#### Remarks.

One pair of strongly sclerotized dorsal furrows is observable only in *H. hirschmannisimilis* Hiramatsu, 1980, but this furrow is Y-shaped in the known species, contrary to the new one, which is not forked. Another difference is the sculptural pattern of the female genital shield: it is smooth in the new species, whereas that of the other species is ornamented. The V-shaped, long, strongly sclerotized ventral furrow is present in the new species, whereas *H. hirschmannisimilis* has two short furrows (reaching from the caudal margin to the anal opening) on the ventral idiosoma.

### Hutufeideria sumatraensis sp. nov.

Taxon classificationAnimaliaEchinostelialesUropodidae

29727988-B4EE-5732-AFB2-163F630655B5

https://zoobank.org/C817E613-2CD2-4625-A59D-DA80EDFAA258

Figs 4, 5, 6

#### Material examined.

***Holotype***. • Female, Indonesia, Sumatra, West-Sumatra Province, Mt. Merapi, c. 15 km of Bukittinggi, 0°23'32"S, 100°26'54"E, 1650–1700 mm, hillforest, 04. June 2006. A. Schulz, coll. ***Paratype***. • One female, same data as holotype.

#### Description.

**Female**. Length of idiosoma 780–785, width 525–531 (*N* = 2). Shape oval, dorsally domed and strongly sclerotized.

**Figure 4.**
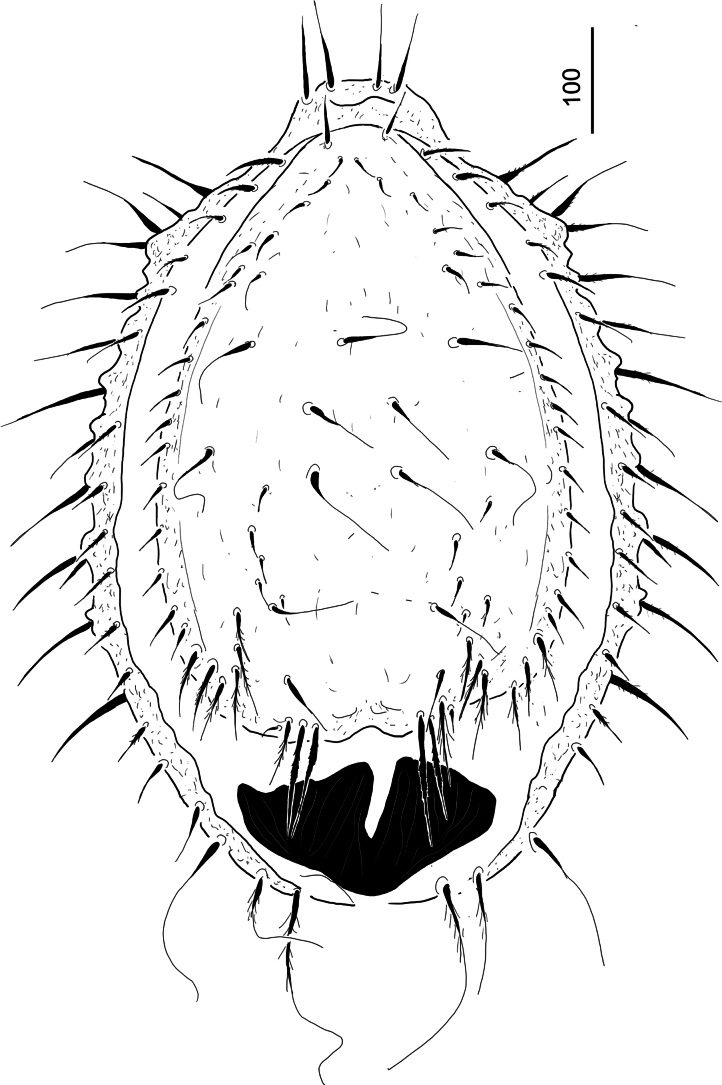
*Hutufeideria sumatraensis* sp. nov., dorsal view of female holotype.

***Dorsal idiosoma*** (Fig. 4). Dorsal and marginal shields completely separated. Majority of dorsal setae on central area long (c. 76–95) and smooth, five pairs of short (c. 24–27) smooth setae situated on laterocentral area of dorsal shield. More than 15 pairs of smooth and short (c. 28–31) setae situated on margins of dorsal shield. Six pairs of pilose (c. 60–78) and two pairs of serrate and robust (c. 115–120 long) setae situated on caudal area of dorsal shield. Dorsal shield without sculptural pattern. Surface of marginal shield smooth and bearing c. 15 pairs of short (c. 38–49) and marginally pilose setae, 12 pairs of long (c. 87–100) and pilose setae on anterior and central area and three pairs of extremely long (c. 120–220) pilose setae on caudal area of marginal shield. Postdorsal shield poorly visible, covered by a large, weakly sclerotized and deeply incised membranous caudal appendix.

**Figure 5.**
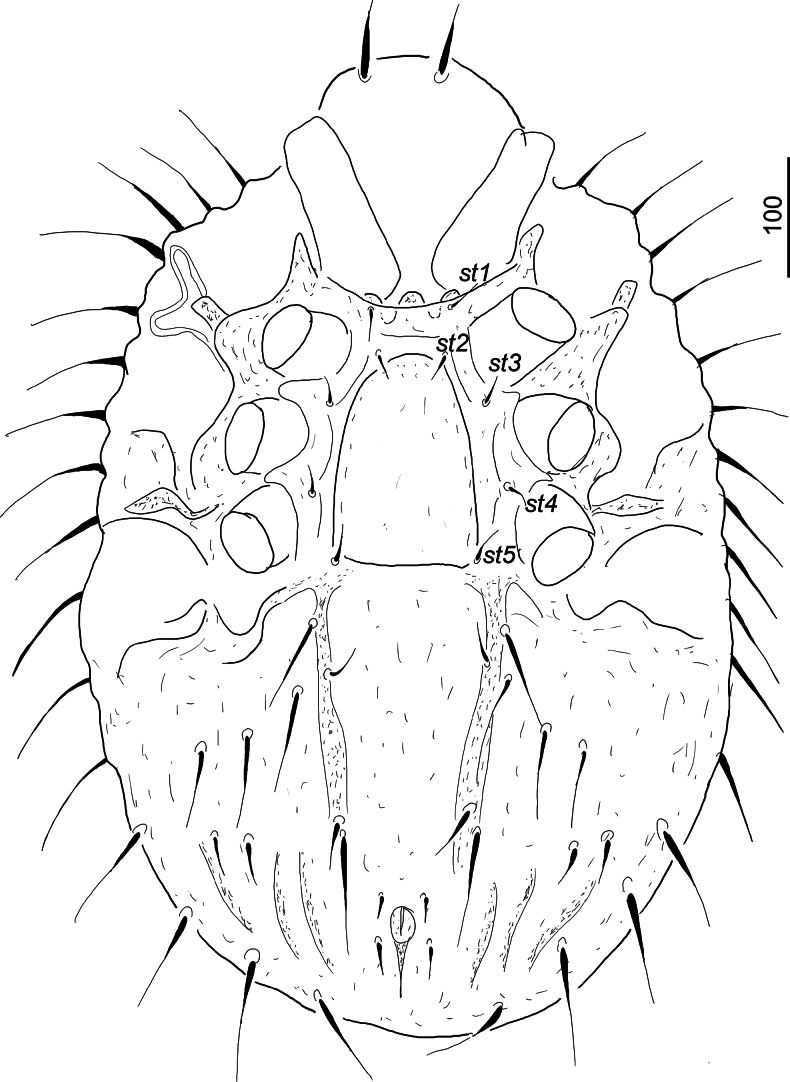
*Hutufeideria sumatraensis* sp. nov., ventral view of female holotype.

**Figure 6.**
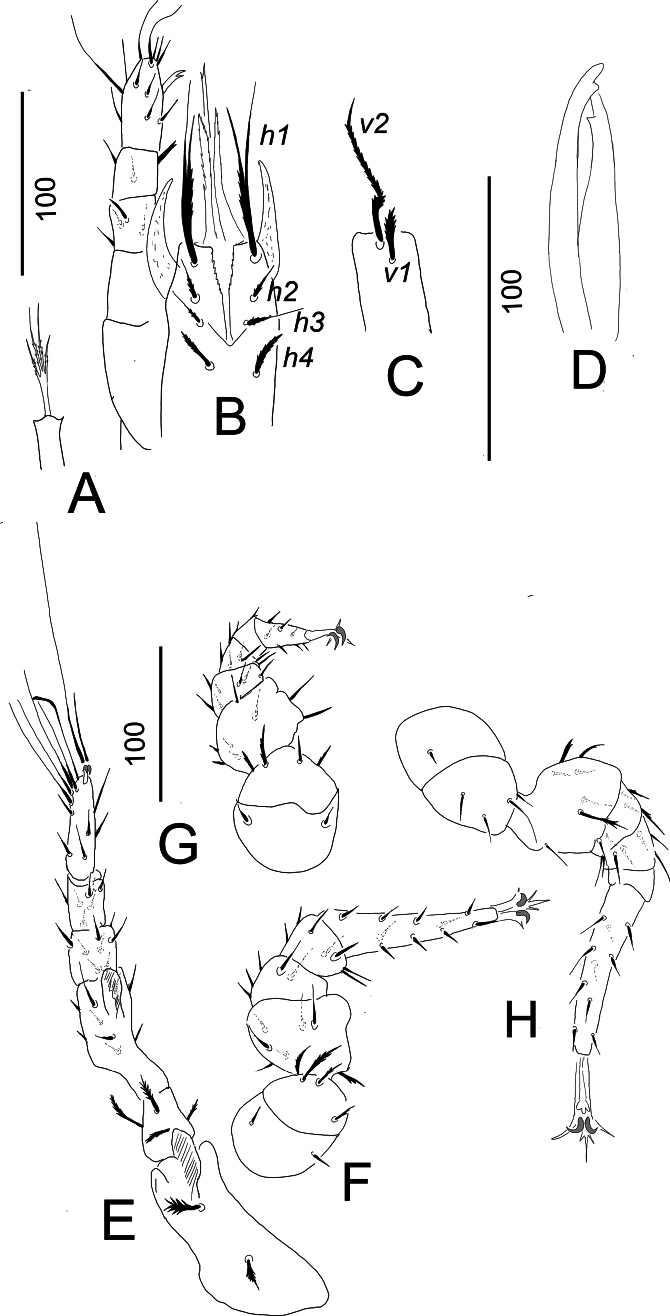
*Hutufeideria sumatraensis* sp. nov., female holotype. **A**. Tritosternum; **B**. Ventral view of gnathosoma; **C**. Ventral view of palp trochanter; **D**. Chelicera; **E**. Ventrolateral view of leg I; **F**. Ventrolateral view of leg II; **G**. Ventrolateral view of leg III **H**. Ventrolateral view of leg IV.

***Ventral idiosoma*** (Fig. 5). Surface of sternal shield smooth, its anterior margin undulate. Five smooth and needle-like sternal setae present. Setae *st1* (c. 26–30) situated close to anterior margin of sternal shield, *st2* (c. 21–24) close to anterior margin of genital shield, *st3* (c. 25–31) at level of posterior margin of coxae II, *st4* (c. 25–27) at level of anterior margin of coxae IV, *st5* (c. 30–32) situated close to basal edge of genital shield. Ventral shield with one pair of central and long and three pairs of lateral and short longitudinal strongly sclerotized furrows. Ventral shield without sculptural pattern. All ventral setae smooth, five pairs of ventral setae long (c. 80–97), the other four pairs short (c. 20–38). Two pair of smooth adanal setae short (c. 20–22), postanal seta absent. Anal opening rounded, 32–34 long and 20–22 wide. Pedofossae deep and their surface smooth, without separated furrows for tarsi IV. Genital shield linguliform (c. 180–187 long and 120–126 wide at level of *st4*), anteriorly rounded, its surface without sculptural pattern. Stigmata situated between coxae II and III, peritremes M-shaped. Tritosternum with narrow basis, laciniae subdivided into four pilose branches (Fig. 6A).

***Gnathosoma*** (Fig. 6B). Corniculi long and horn-like, without lateral spines. Internal malae longer than corniculi and marginally pilose. Hypostomal setae *h1* (c. 95–99 µm) subdivided into two branches of which one smooth and other one marginally pilose, *h2*, *h3* and *h4* marginally serrate and short (c. 20–32 µm). Epistome apically pilose. Palp trochanter with a short (*v1*) and a long (*v2*) marginally serrate setae (Fig. 6C), setae on femora serrate, other setae on palp smooth. Apical part of chelicerae with one-one tooth on both digits, other part of chelicerae not visible without breaking the idiosoma (Fig. 6D).

***Legs*** (Fig. 6E–H). Majority of setae on legs smooth, but some setae pilose. Claw of leg I smaller than on others, flap-like prolongation visible on femora of leg I–IV. Length of legs (from the basis of coxae to the tip of tarsi): leg I 380–394, leg II 280–284, leg III 260–271, leg IV 310–317.

**Male**, nymphs and larvae unknown.

#### Etymology.

The name of the new species refers to the island where it was collected.

#### Remarks.

The smooth dorsal and ventral shields and short and long setae situated on the dorsal shield are visible only in *H. deliciosa* Hiramatsu, 1978, but the dorsal setae on the central area are smooth in the new species, and two pairs are apically pilose in *H. deliciosa*. The strongly sclerotized furrows on the ventral shield are present in the new species but absent in the known species. On the other hand, the second pair of adanal setae is smooth in the new species versus pilose in the previously described one.

### Hutufeideria sarawakensis sp. nov.

Taxon classificationAnimaliaEchinostelialesUropodidae

2A3C1333-7DFD-573D-BBDE-EDE56F09ED85

https://zoobank.org/8E1D1C9F-8987-45CA-A08B-C022DB9FD783

Figs 7, 8, 9

#### Material examined.

***Holotype***. • Female, Sar-87/76. Malaysia, Sarawak, Bako National Park, Jalan Lintang, prélèvement de sol dans les angles formés par les contreforts de *Austrobuxus nitidus* [=*Longetia malayana*] (Euphorbiaceae), 30 m, 11. December 1987, B. Hauser coll. (Berlese à Genève, Suisse). ***Paratype***. • One male, same data as holotype.

#### Description.

**Female**. Length of idiosoma 630, width 370 (*N* = 1). Shape oval, dorsally domed and strongly sclerotized.

**Figure 7.**
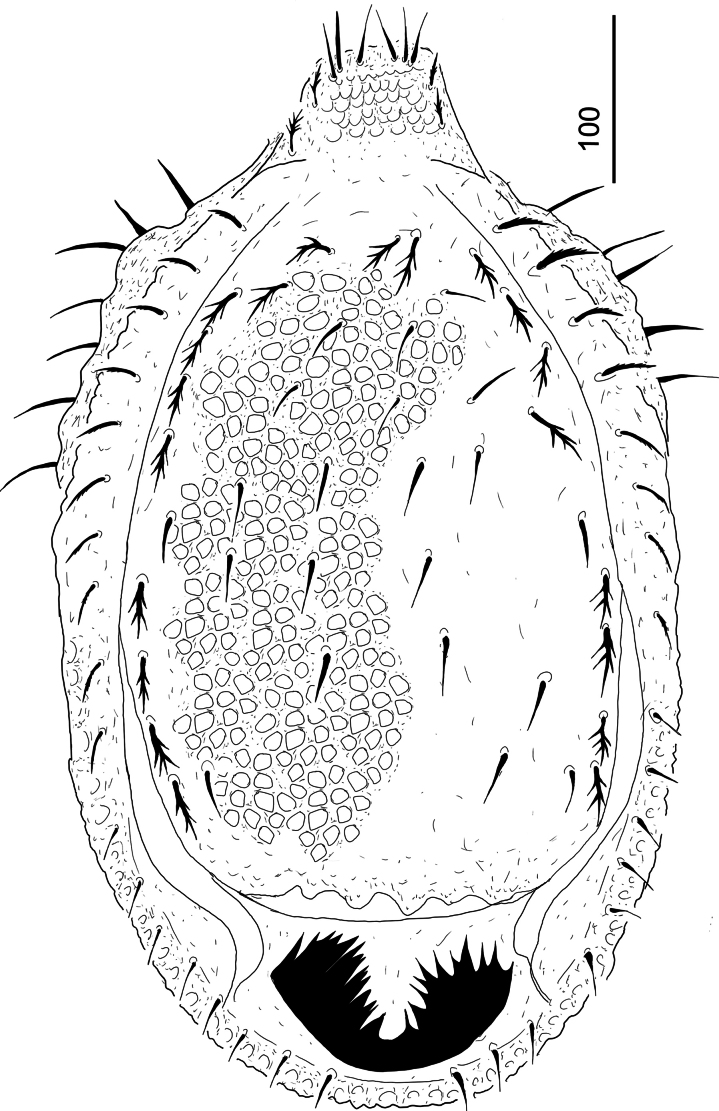
*Hutufeideria sarawakensis* sp. nov., dorsal view of female holotype.

***Dorsal idiosoma*** (Fig. 7). Dorsal and marginal shields fused anteriorly. Dorsal setae on central area c. 25–38 long and smooth. Setae on anterior and central margins of dorsal shield pine-tree shaped and 38–40 long. Dorsal shield covered by irregular pits (8–15 × 9–10). Posterior margin of dorsal shield undulate. Surface of marginal shield with some oval pits on caudal and lateral areas. Marginal shield bears c. 19 pairs of c. 25–42 long and marginally serrate setae. Postdorsal shield large, covered by a large, weakly sclerotized and deeply incised membranous caudal appendix (c. 65–70 long), its anterior margins strongly serrate.

**Figure 8.**
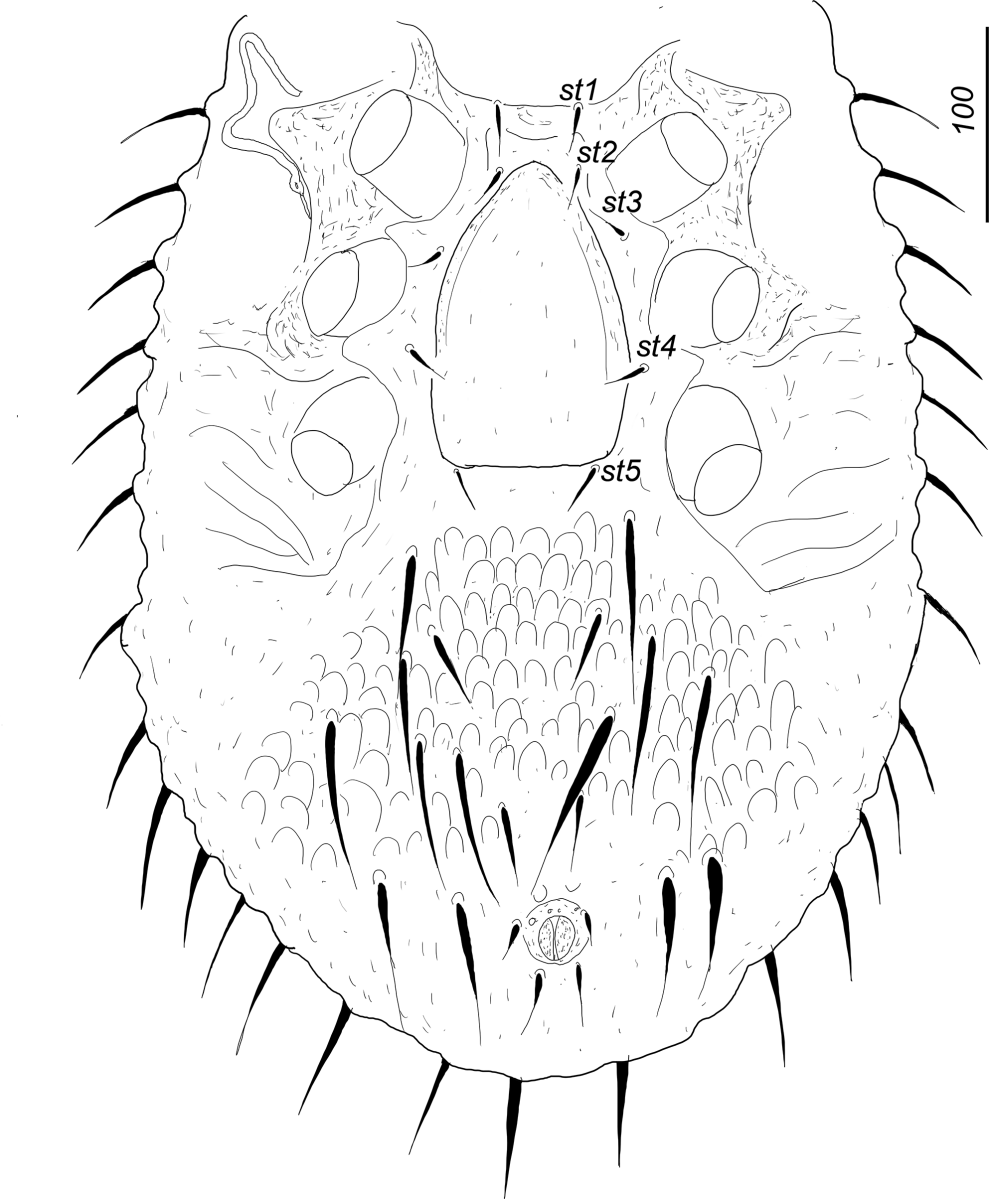
*Hutufeideria sarawakensis* sp. nov., ventral view of female holotype.

***Ventral idiosoma*** (Fig. 8). Surface of sternal shield smooth, its anterior margin straight. Five smooth and needle-like sternal setae present. Setae *st1* (c. 25–26) situated close to anterior margin of sternal shield, *st2* (c. 23–24) close to anterior margin of genital shield, *st3* (c. 24–25) at level of posterior margin of coxae II, *st4* (c. 25–27) at level of posterior margin of coxae III, *st5* (c. 25–26) situated close to basal line of genital shield. Ventral shield covered by tile-like sculptural pattern. All ventral setae smooth, six pairs of ventral setae long (c. 75–96), the other two pairs short (c. 37–43). Two pairs of smooth adanal setae, first pair shorter (c. 20–21) than second pair (c. 33–35), postanal seta absent. Anal opening rounded, 21 long and 20 wide. Pedofossae deep and their surface smooth, without separated furrows for tarsi IV. Genital shield scutiform (c. 157 long and 95 wide at level of *st4*), anteriorly slightly peaked, its surface without sculptural pattern. Stigmata situated between coxae II and III, peritremes M-shaped.

***Tritosternum and parts of gnathosoma***. Not visible, covered by coxae I.

**Figure 9.**
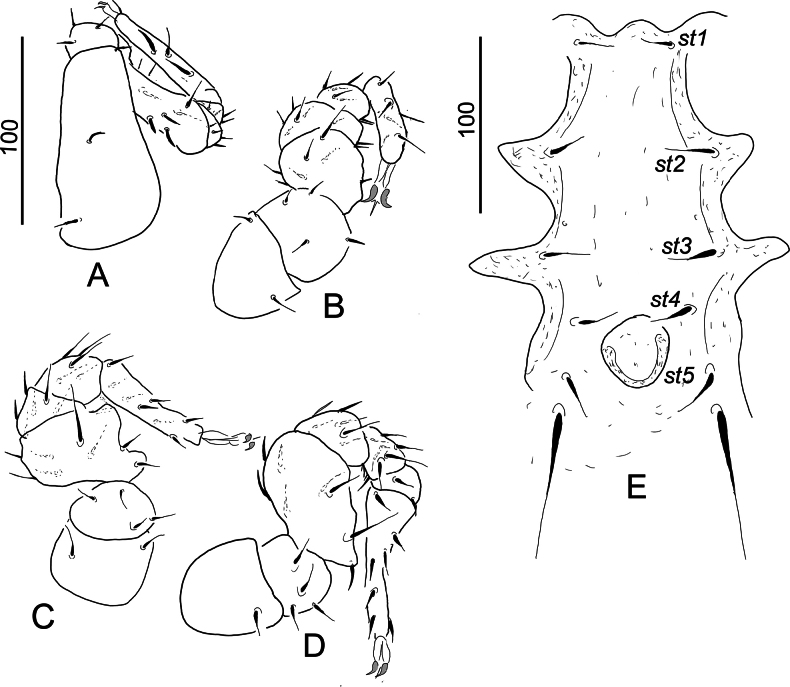
*Hutufeideria sarawakensis* sp. nov., female holotype (**A–D**), male paratype (**E**). **A**. Ventrolateral view of leg I; **B**. Ventrolateral view of leg II; **C**. Ventrolateral view of leg III; **D**. Ventrolateral view of leg IV; **E**. Intercoxal area.

***Legs*** (Fig. 9A–D). Majority of setae on legs smooth, but some setae pilose. Claw of leg I smaller than on others, flap-like prolongation visible on femora of leg I–IV. Length of legs (from the basis of coxae to the tip of tarsi): leg I 275–278, leg II 203–205, leg III 253–254, leg IV 244–246.

**Male**. Length of idiosoma 625, width 367 (*N* = 1). Shape oval, posterior margin rounded.

***Dorsal side***. Ornamentation and chaetotaxy as in female.

***Ventral side***. Ornamentation and chaetotaxy of ventral shield as in female. Sternal shield without sculptural pattern, the positions of five pairs of smooth and c. 20–28 long sternal setae as in Fig. 9E. Genital shield rounded (40 long and 39 wide) and situated between coxae IV.

Nymphs and larvae unknown.

#### Etymology.

The name of the new species refers to the island where it was collected.

#### Remarks.

The new species has pine-tree-like setae on the caudal part of the dorsal shield; this character state is otherwise observed only in *H. virtuosa* Hiramatsu, 1983. The most important differences between these two species are as follows: (1) the weakly sclerotized membranous caudal appendix is rounded in the known species versus apically serrate and bilobed in the new species; (2) the pits on the dorsal shield are oval in *H. virtuosa* versus irregular in the new species, and (3) the ventral setae are short in the known species versus very long in the new species.

##### Zoogeographical notes

The known hutufeideriid species occur in the Austral-Asian region. The northern border of the family seems to be Japan, and the southern border is Australia. Based on available distribution data, the biodiversity hotspot for this family appears to be Southeast Asia; however, we have very little information about this family, and we should expect that many new species will be discovered in Southeast Asian soils in the near future.

## Supplementary Material

XML Treatment for Hutufeideriidae

XML Treatment for Hutufeideria

XML Treatment for Hutufeideria perakensis

XML Treatment for Hutufeideria sumatraensis

XML Treatment for Hutufeideria sarawakensis

## Additional information

### Artificial Intelligence (AI) use

The authors accept full responsibility for the content of the manuscript, including the disclosure of any use of AI.

No AI tools were used in the preparation of this manuscript.

### Author ORCIDs

J. Kontschán https://orcid.org/0000-0001-8274-4238

R. Ábrahám https://orcid.org/0000-0002-9062-1382

S.G. Ermilov https://orcid.org/0000-0002-0913-131X

### Data availability

All of the data that support the findings of this study are available in the main text.
